# Analysis of platelets from a diet-induced obesity rat model: elucidating platelet dysfunction in obesity

**DOI:** 10.1038/s41598-020-70162-3

**Published:** 2020-08-04

**Authors:** María N. Barrachina, Luis A. Morán, Irene Izquierdo, Felipe F. Casanueva, María Pardo, Ángel García

**Affiliations:** 1 Platelet Proteomics Group, Center for Research in Molecular Medicine and Chronic Diseases (CIMUS), Universidade Santiago de Compostela, Avda de Barcelona s/n, 15782 Santiago de Compostela, Spain; 2 Instituto de Investigación Sanitaria de Santiago (IDIS), Santiago de Compostela, Spain; 3 CIBER de Fisiopatología Obesidad y Nutricion (CIBERobn), Instituto Salud Carlos III, Santiago de Compostela, Spain; 4 Department of Medicine, Universidade de Santiago de Compostela, Complexo Hospitalario Universitario de Santiago (CHUS), Santiago de Compostela, Spain; 5 Grupo Obesidómica, Instituto de Investigación Sanitaria de Santiago (IDIS), Xerencia de Xestión Integrada de Santiago (XXS), Santiago de Compostela, Spain

**Keywords:** Obesity, Platelets

## Abstract

Obesity is one of the main health problems in industrialized countries. The contribution of multiple factors developed in obesity can hardly be modeled in vitro. In this context, the development of animal models mimicking human obesity could be essential. The aim of the present study was to compare platelets from a diet-induced obesity (DIO) rat model with their lean control group in order to elucidate platelet dysfunction mechanisms in obesity and correlate the results with previous data from morbid obese patients. In parallel, we also established a blood collection and platelet isolation methodology to study the DIO rat model at biochemical and functional level. Optimal blood collection was obtained from vena cava and platelet isolation was based on a serial of centrifugations avoiding platelet activation. Our results show that the DIO rat model simulate obesity pathologically since weight gain, fasting glucose and platelet counts are increased in obese rats. Interestingly, platelet levels of the active form of Src (pTyr^419^) showed a tendency to increase in DIO rats pointing towards a potential dysfunction in Src family kinases-related signalling pathways in obesity. Moreover, platelets from DIO rats adhere more to collagen compared with the control group, pointing towards Glycoprotein VI (GPVI) as one of the dysregulated receptors in obesity, in agreement with our recent studies in humans. These results confirm that obesity, in line with human studies, present a platelet dysregulation, and highlight the relevance of considering novel antithrombotic drug targets in these patients, such as GPVI.

## Introduction

The global obesity epidemic represents one of the biggest health problems in industrialized countries. It is well known that obesity-associated alterations constitute relevant risk factors for several diseases, including diabetes, cancer, arthritis, high blood pressure, and cardiovascular diseases (CVD). In fact, the link between obesity and CVD has been repeatedly reported with the chronic exposure to a pro-inflammatory and pro-thrombotic state in obese individuals in which platelets have a pivotal role^1^. Moreover, recent studies described the pathogenetic role of platelet hyperactivation and reduced sensitivity to antiaggregating therapy in obesity^1^. Nonetheless, the exact mechanisms by which adiposity induces platelet dysfunction remain poorly investigated^2^.

The contribution of multiple factors developed in obesity can hardly be modeled in vitro and that is why using animal models could be critical for this purpose. Whilst animal models cannot thoroughly replicate human obesity, they are a powerful tool to develop new therapeutic approaches for preventing and treating the pathology. Over the last 2 decades, many groups have employed a established animal model, diet-induced obesity (DIO) model, to understand the development and persistence of obesity, as well as the metabolic complications that accompany the excess weight^3^. To perform this model, rats are into a regimen of a high-fat diet (more than 40% of fat content) during several weeks, leading to obesity, hyperglycemia, hypertriglyceridemia and hyperleptinemia. Today, this animal model is recognized as one of the most widely used models to mimic the pathophysiology of human obesity and metabolic syndrome^4^. Nevertheless, there are only few studies that investigated platelet dysfunction in an obese rat model to date^5–8^. In this context, the first goal of the present study was to establish a standardized protocol for rat blood collection and platelet isolation to enable data comparison between research laboratories and transfer the findings to human clinical studies.

We have recently performed some studies with platelets from morbid obese patients, comparing them with their lean-matched controls, where we found an alteration in Src family kinases (SFKs) signalling pathways, especially Glycoprotein VI (GPVI) in obesity^9,10^. In line with the above, the second goal of the present study was to characterize the impact of obesity in DIO rat platelets and try to correlate their biochemical parameters with those we previously obtained in humans. In this way, both the animal model and our previous clinical data would be cross validated helping at the same time to elucidate platelet dysfunction in obesity.

## Results

### The DIO rat model mimics the pathological characteristics of obesity

**Figure 1:**
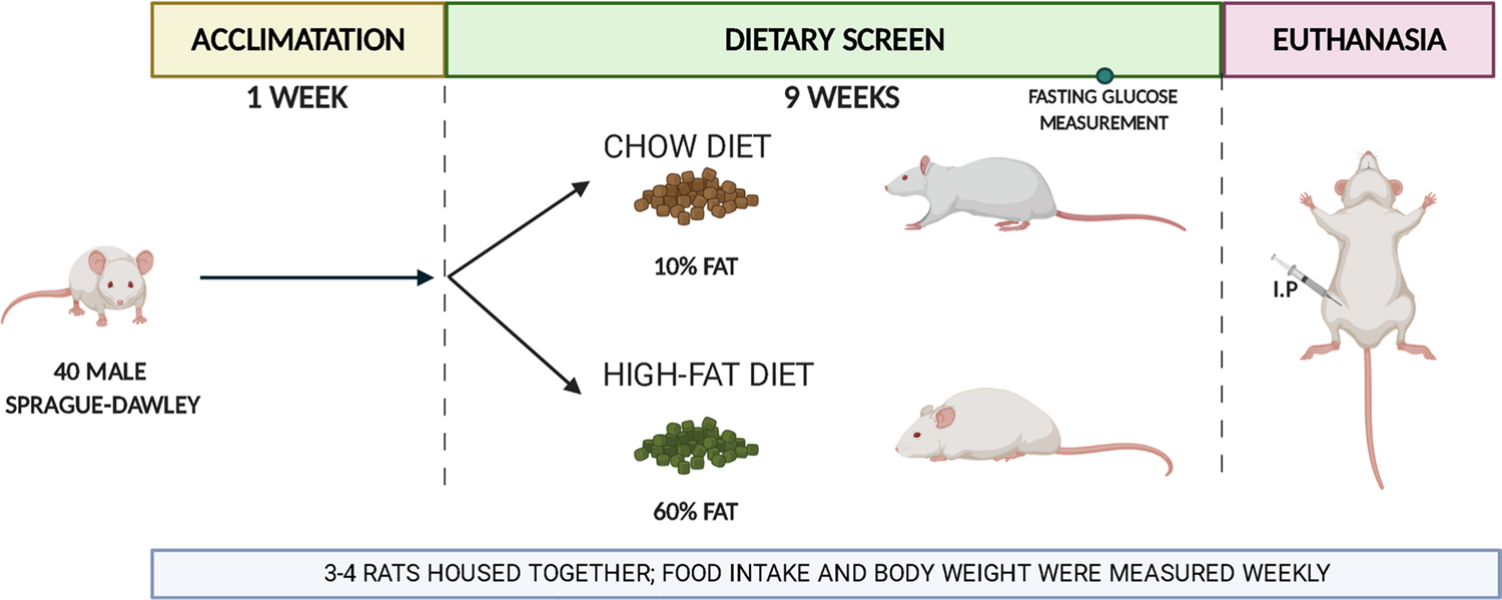
Detailed time-schedule to set up a DIO rat model for platelet studies. The time-schedule for the rat procedure was divided in three steps: acclimation, dietary screen and euthanasia. Created with Biorender.com.

40 adults Sprague–Dawley rats were provided free access to standard diet (10% fat) for a 1‐week baseline period. Following this phase, they were separated randomly into two experimental groups (n = 20/per group): fed with standard diet or HFD for 9 weeks (Fig. 1).

**Figure 2:**
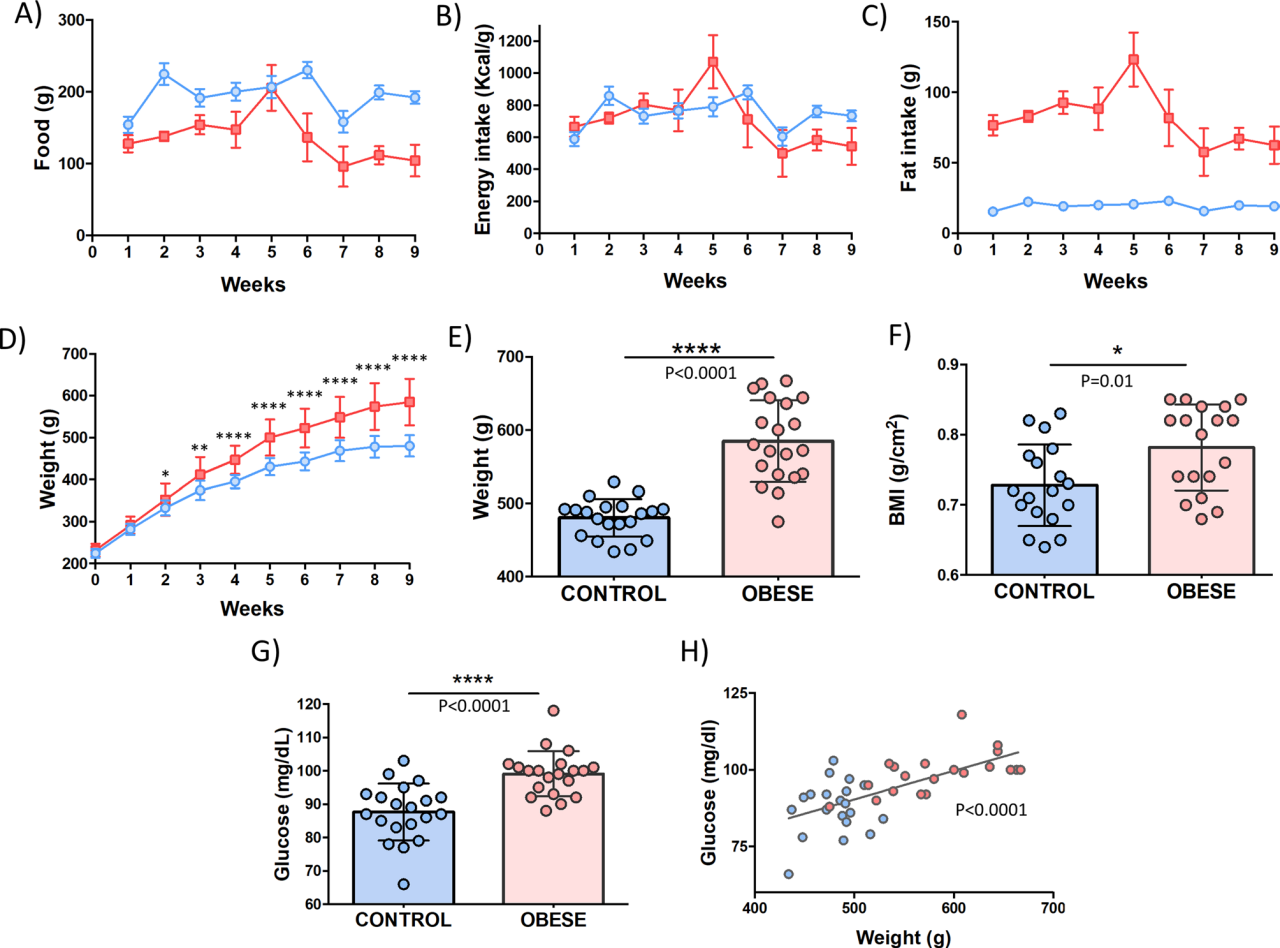
Analysis of parameters related to the diet-induced obesity rat model. Food intake (**A**), energy intake (**B**), fat intake (**C**) and weigh gain (**D**) during the procedure. (**E**) Final weight and (**F**) BMI of both groups at the end of the procedure. (**G**) Fasting glucose levels. (**H**) Positive correlation between fasting glucose levels and weight. Controls (n = 20) and DIO rats (n = 20) are represented in blue and red color, respectively.

The DIO rat and lean-matched control groups were fed at libitum during the dietary period (9 week) (Fig. 2a). In terms of energy intake, no differences were found between groups (Fig. 2b). Interestingly, the amount of fat intake in the DIO group decreased after the 5th week of the procedure (Fig. 2c).

As expected, the HFD produced a significantly increased body weight when compared to controls during the procedure (Fig. 2d). After 9 weeks of different dietary pattern, significant differences in the distribution of body weight were found in the DIO group (Fig. 2e,f). Moreover, others anthropometrical parameters related to the obesity-associated pathogenesis were measured. This data showed that the DIO rat model had more thoracic and abdominal circumference compared to their lean-mated group (Table 1). Furthermore, fasting glucose was also tested presenting higher levels in the HDF-feed model and correlating positively with the final weight (Fig. 2g,h).

**Table 1:** Weight gain, fasting glucose and anthropometric parameters of Sprague–Dawley rats fed either with standard or high-fat (HF) diet for 9 weeks.

Parameters	Control (n = 20)	Obese (n = 20)
Weight (g)*	480.30 ± 25.49	584.80 ± 55.51
Fasting glucose (mg/dL)*	87.65 ± 8.54	99.10 ± 6.80
Abdominal circumference (cm)*	18.67 ± 1.45	21.78 ± 1.66
Thoracic circumference (cm)*	17.53 ± 1.17	19.00 ± 0.84

*p < 0.05, data represent mean ± standard deviation.

### Establishment of a platelet isolation method to study rat platelets

**Figure 3:**
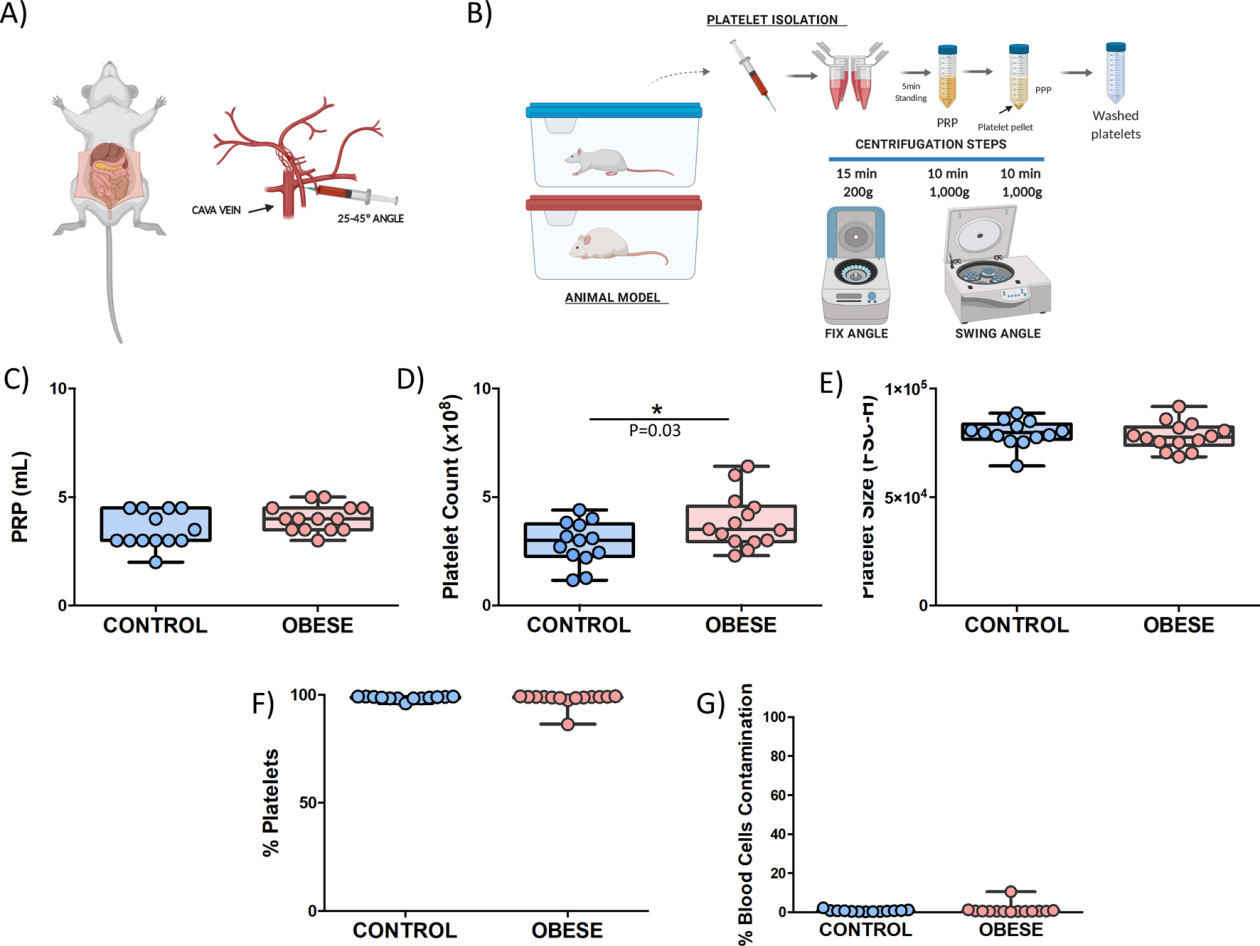
Platelet isolation protocol and quality-associated parameters. (**A**) Blood collection was done via vena cava with a 19G needle syringe. (**B**) Platelet isolation protocol was based on a serial of centrifugations. (**C**) Levels of PRP and (**D**) platelet counts measured during the platelet isolation protocol. (**E**) platelet size parameters; (**F**) % of pure platelets; and (**G**) % of blood cell contaminations. Statistical analysis was done by unpaired t-test. Runs were done in duplicate. Controls (n = 13) and DIO rats (n = 14) are represented in blue and red color, respectively. (**A**, **B**) were created with Biorender.com.

Choosing adequately the best option for blood collection and platelet isolation depending on the planned experiment is an important issue. In the case of our study, protocols were designed based on several guidelines developed for mice platelets that we modified and adapted to our rat model (Fig. 3a,b)^11,12^.

In order to evaluate the efficiency and reproducibility of the procedure, a variety of different quality parameters was successfully measured in a total number of 14 DIO rats and 13 lean-matched controls.

As shown in Fig. 3c, platelet-rich plasma (PRP) levels were almost the same between groups pointing towards a high degree of reproducibility in the methodology. Additionally, in terms of platelet-related parameters, platelet counts were significantly higher in the DIO rat model compared to control rats (Fig. 3d). Moreover, no positive correlation was found between platelet counts and total weight and BMI (Supplementary Fig. 1a,b). Further, no significantly differences were found between groups regarding platelet size and there was no positive correlation between the latter and weight (Fig. 3e, Supplementary Fig. 1c).

Apart from the parameters mentioned above, contaminations with other blood cells is an important issue to consider. According to the results, the percentage of “pure” platelets was more than 99% meaning that we established a platelet isolation method where less than 1% belongs to others blood cells contaminants (Fig. 3f,g).

To sum up, the global data showed that the methodology set up shows a high degree of reproducibility between groups allowing collecting more than 99% of pure platelets in the sample.

### Active Src (pTyr^419^) levels are increased in DIO platelets

**Figure 4:**
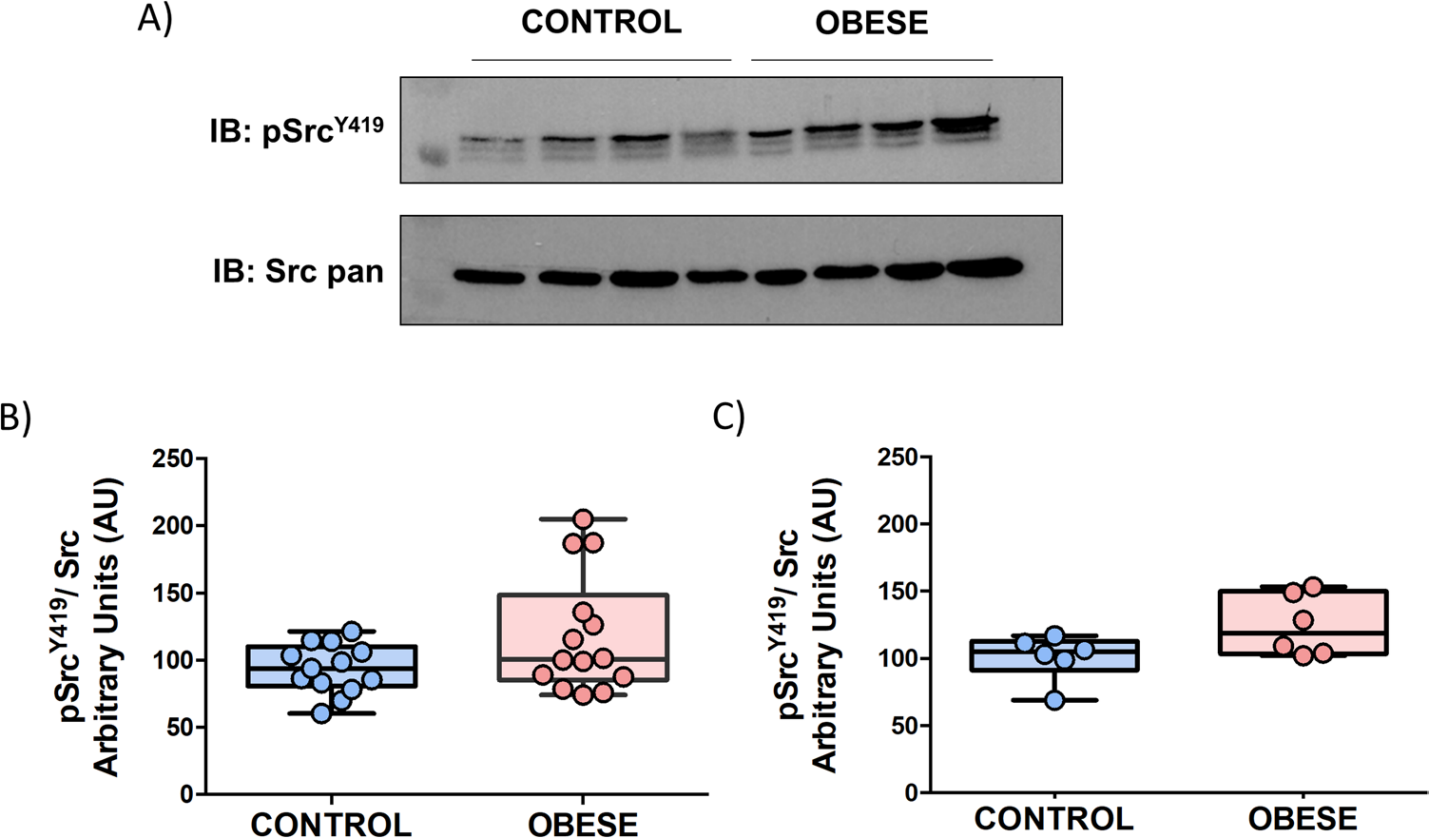
DIO rat platelets show platelet signalling alterations related to SFKs-mediated pathways. (**A**) 1D-Western blot analysis of Src-pTyr^419^ and Src pan protein expression levels in platelets from individual samples (13 lean-matched controls and 14 DIO rats). (**B**) Densitometry graphs showing the mean values ± SD of Src (Tyr 419) corrected by Src. Statistical analysis was done by unpaired *t* test (*p* = 0.06). (**C**) Densitometry graphs showing the mean values ± SD of Src (Tyr 419) corrected by Src in a subgroup of animals (extreme weight and high levels of glucose, 6 DIO and 6 controls). Statistical analysis was done by unpaired *t* test (*p* = 0.07). Images are representative of the results obtained and show samples distributed in one gel (Supplementary Fig. 2). *IB* immunoblot.

To investigate platelet dysfunction in our obese rat model, we analyzed the platelet expression levels of the active form of Src^Y419^ by western blotting assays. Src and other members of the SFKs are tightly regulated by tyrosine phosphorylation. Full catalytic activity of Src requires phosphorylation of tyrosine 419, which is located in the catalytic domain. By using a specific anti-Src (pTyr419) antibody, a cohort of 14 DIO rats and 13 lean-matched controls were compared. According to the results, there is a clear tendency towards increased Src^Y419^ levels in the obese group (fold change = 1.3) (Fig. 4a,b).

Furthermore, we also analyzed a subgroup of animals with extreme weight and high glucose levels (comparing 6 rats per group) trying to resemble the human comparison between morbid obese patients and slim controls. In line with the previous results, there is also a tendency towards increased Src^Y419^ levels in the obese group (Fig. 4c).

These data are in line with the results previously obtained in morbid obese patients pointing towards a potential dysfunction in SFKs-related signalling pathways^9^.

### Platelet adhesion to collagen is increased in DIO rats

**Figure 5:**
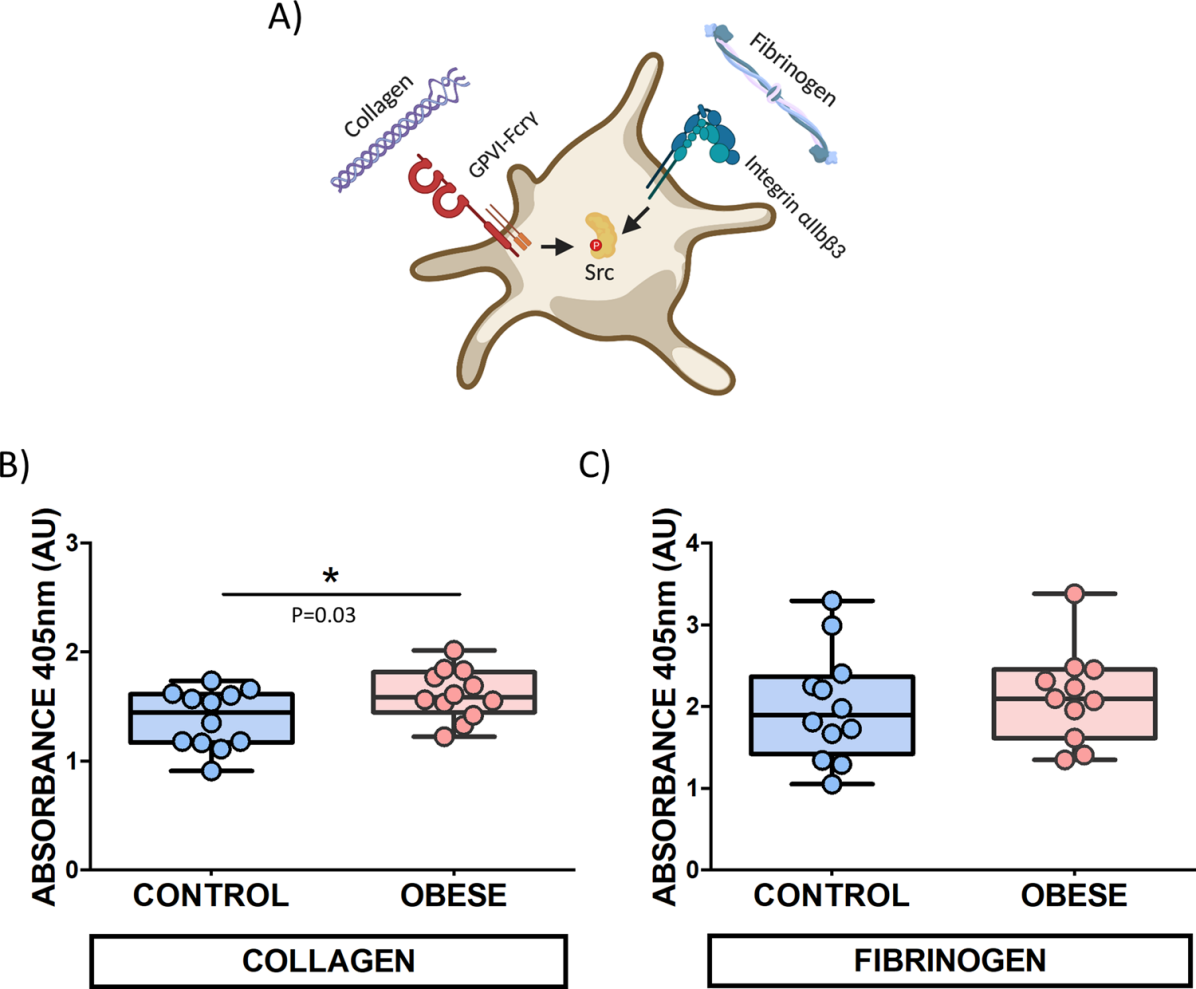
DIO rat platelets show higher adhesion to collagen. (**A**) Schematic picture of the primary SFKs-related receptors, GPVI and integrin αIIbβ3. Washed platelets from both groups adhered on plates coated with (**B**) collagen (5 µg/mL) and (**C**) fibrinogen (100 µg/mL). Absorbance 405 nm was measured. Runs were done in triplicate. Statistical analysis was done by unpaired *t* test, collagen (*p* = 0.03) and fibrinogen (*p* = 0.6) *p < 0.05. (**A**) was created with Biorender.com.

The above results suggested a potential platelet dysfunction related to SFK-mediated signalling pathways. To further explore these platelet alterations in our rat model, functional studies using static adhesion assays were performed. Plates were coated with collagen and fibrinogen in order to better understand the impact of obesity on primary SFKs-related receptors, GPVI and integrin αIIbβ3, respectively (Fig. 5a).

A comparative analysis between 12 obese rats and their 12-matched controls were done. Increased levels of platelet adhesion to collagen-coated plates were observed in the obese group (Fig. 5b). Nevertheless, no significant differences between DIO and control group regarding platelet adhesion to fibrinogen were found (*p* = 0.6) (Fig. 5c).

## Discussion

The aim of the present study was to establish a DIO rat model in obesity-related platelet research and to understand better the pathological mechanisms related to platelets in obesity elucidating new drug targets, drug resistance, and biomarker discovery in this pathology. The main findings of this study are: (1) establishment of a reproducible blood collection and platelet isolation method for platelet functional and biochemical studies in rats; (2) the active from of Src (pTyr419) displays an increase tendency in DIO rat platelets; (3) platelets from DIO rats showed more adhesion to collagen compared to lean controls.

In endocrinology-related research, there are several animal models that are suitable for studying obesity. However, it is important to choose adequately the best model depending on the main goals of your planned study. Indeed, the animal models associated to obesity research can be classified into two main different models: developing obesity due to monogenic mutations (e.g. Zucker fatty rats) or diet-induced animal model (DIO rat). In the case of monogenic mutation (spontaneous or engineered), this model can be extremely useful to study specific mechanistic aspects of eating controls. In 1961, Zucker et al.^13^ observed an obese phenotype in rats due to an autosomal recessive spontaneous mutation affecting the leptin receptor gene. These mutations are present in a small number of obese people becoming this model less suitable to embrace “common” obesity from the human population^14^. However, the other main obesity-related model, DIO rats, (polygenic animal model) is known to replicate the mechanisms of promoting obesity and metabolic syndrome in humans. Indeed, these animals are usually used to study the role of diet, physiopathology and etiology of the disease, as well as pharmacological tests^15^.

In humans, weight gain due to prolonged exposure to fatty diets over a long period of time has been reported in several studies^16,17^. In this context, the DIO rat model allows to characterize and monitor the contribution of HFD to the body weight gain mimicking pathological characteristics of human obesity^18^. This model also allowed us to correlate data from the animal model with data obtained in obese patients, contributing to a better understanding of platelet dysfunction in obesity.

Employing the most appropriate procedure for blood collection and platelet preparation in animal models, especially rodents, is essential for in-vitro assays. Unfortunately, preparation protocols described in the literature are inconsistent and describe many collection methods, isolations steps, materials and anticoagulant buffers^19^. Thus far, only a few studies tried to define the most suitable procedure to exclude platelet pre-activation during the sampling and preparation procedure^11,12,19^. Studying platelets from a rat model has been poorly investigated. However, and due to the platelets similarities with mice, we combined several practical guides previously tested in mice in order to establish our procedure^11,12^.

At the end of the feeding procedure, rats were anesthetized with ketamine/xylazine cocktail intraperitoneally for blood collection. In terms of blood collection, the most common sites for terminal procedure in rodents are heart puncture (HP) and VCP. According to Jirouskova et al.^19^ HP produces tissue factor-reach heart muscle tearing during blood collection, which could lead to thrombin generation and platelet activation. In the case of obese rats, HP has even more drawbacks since the heart is more difficult to identify properly without opening the thorax cavity because of the excess of fatty. On the other hand, VCP is known to be the most recommended blood collection method in terms of the high amount of blood obtained, easy accessibility to the vein and less platelet activation compared with the other methods^11,19^. In line with the data mentioned above, we collected blood via VCP to obtain a high amount of blood with less platelet activation*.*

During the experimental set-up, different needles were used to obtain optimal conditions for blood extraction efficiency and technical handling. Precisely, 21G was the most easily needle used because of its size, gauge and handling. In terms of platelet pre-activation, Pedersen et al. found no differences in platelet pre-activation (less 10%) comparing a 19- and a 21-gauge needle, becoming both suitable for blood sampling^20^.

After the puncture, blood should be collected directly in the presence of an anticoagulant. In platelet-related studies, several anticoagulants are commonly used depending on the goal of the analysis. In general, ethylenediaminetetraacetic (EDTA) acid is used to measure accurate platelet counts and mean platelet volume. In the case of heparin, this is usually used for platelet function assays (whole blood and PRP). Finally, platelet signalling studies conducted in PRP and washed platelets are primarily carried out using sodium citrate as anticoagulant. In the case of the present procedure, we combined two anticoagulants (sodium citrate and ACD) during platelet isolation. This methodology is particularly useful for phosphorylation studies because it allows a high concentration of platelets minimizing unwanted activation by the addition of prostacyclin PGI_2_^21^.

The platelet isolation procedure employed in the present study was based on a serial of centrifugation steps avoiding other blood cells contamination and spontaneous platelet activation. The centrifugation steps were adapted using a practical guide for mice since the platelet size of both rodents are practically similar^22^. Moreover, the efficiency and reproducibility among samples during the isolation protocol is an important issue to consider. In this context, our methodology allowed a high degree of reproducibility between groups allowing more than 99% of pure washed platelets.

In terms of platelet-related parameters, platelet counts, and platelet size are both commonly associated with platelet dysfunction^23–26^. Indeed, both parameters are directly related with cardiovascular risk; indeed there is a positive correlation between platelet counts and other anthropometric parameters such abdominal circumference, and fat distribution^27^. In our case, we found that platelet counts were significantly higher in the DIO model compared to control rats. However, no differences were found on platelet size among groups. In this context, using obese non-diabetic rats (Zucker fatty model) Hernández-Vera et al.^7^ postulated alterations in platelet count and increased platelet size in this model.

We recently reported that platelets from obese patients showed a potential alteration in SFK-related signalling pathways, in particular the collagen receptor GPVI^9^. Src is a 60-kDa nonreceptor tyrosine kinase which is involved in transmitting activation signals from different platelet surface receptors, which are essential for thrombus growth and stability (GPVI, integrin αIIbβ3, CLEC-2, among others)^28^. Phosphorylation of the residue Tyr^419^ is necessary to reach full catalytic activity of Src. In line with our data in humans, we found a clear tendency of increased pSrc^Y419^ levels in DIO rat platelets, although the interindividual variability is high.

GPVI and integrin αIIbβ3 platelet stimulation lead to activation of Src through autophosphorylation at position Tyr^419^^28^. Interestingly, we demonstrated that platelets from DIO rats display increased levels of adhesion to collagen-coated plates pointing towards an alteration in GPVI signalling. In this context, a study carried out by Kreuts et al.^29^ demonstrated that obese pigs also had higher platelet aggregation in response to collagen than lean pigs. In humans, Leite et al.^30^ also showed that platelets from obese patients were hyperreactive in response to collagen. Indeed, as mentioned above, our group has recently showed that platelets from obese patients have higher GPVI surface levels and higher platelet aggregation in response to collagen-related peptide (CRP) and collagen^9^.

The overall previous data in humans are now reinforced by our animal model confirming a hyperactivation of GPVI signalling in obesity. In terms of mimicking the pathophysiology of human obesity, the DIO rat model is an optimal choice to transfer the findings to human clinical studies.

The present study has some limitations that should be considered. An efficient blood sampling was not trivial. Thus, although the study was performed with 40 rats, several of them presented difficulties during euthanasia and blood collection. Since the quality of the sample is one of the main considerations for the analysis, these rats’ samples were removed to know for certain that the quality of the study was not going to be affected.

Another limitation was the lack of reagents available to do functional studies. For instance, it would have been of interest to check GPVI platelet surface expression levels by flow cytometry in obese rats but there is no such antibody available for rat GPVI. In addition, sample limitation prevented us from doing additional experiments in parallel, such as immunoprecipitations directly against the validated proteins, or platelet aggregation assays.

In summary, the present study allowed to establish an adequate blood collection and platelet isolation method for DIO rats and contributes to elucidate platelet dysfunction in obesity. The methodological development will enable data comparison between laboratories and a better transfer of the findings to clinical studies. In addition, we show that platelets from DIO rats exhibit more adhesion to collagen-coated plates pointing towards an up-regulation of GPVI signalling in obesity. Overall, the DIO animal model could be a powerful tool to study obesity-related alterations in rat platelets. Indeed, our results confirm that obesity is related to an atherothrombotic risk that should be considered and, in line with our studies in humans, highlight the relevance of further studying GPVI as anti-thrombotic drug target in obesity. We hope that our study will open new lines of investigation to further explore the role of platelets in obesity.

## Material and methods

### Animal model

The study was performed under the procedure with code 15005/2015/003, according to the institutional guidelines and the European Union standards for the care and use of experimental animals. The procedures were approved by the *Consellería de Medio Rural*, *Xunta de Galicia*, and the Animal Care Committee of Santiago de Compostela University (Santiago de Compostela, Spain).

Forty adult Sprague–Dawley male rats weighing 150–199 g and 5 weeks of age were purchased from the animal facilities of the University of Santiago. Rats were kept for 7 days in the animal husbandry to enable acclimatization to the local conditions (temperature (22–24 °C) and light/dark cycle (12 h light, 12 h darkness)). After acclimation, animals were randomly separated into two experimental groups (n = 20/per group): control and DIO models.

Regarding the dietary, DIO animals were fed with 60% high fat diet (HFD) with an energy density of 5.21 kcal/g. The caloric information is as follows: 60% Kcal fat, 20% Kcal protein, 20% Kcal carbohydrate (D12492, Research Diets, NJ). Moreover, the 60% HFD contains the following ingredients: Casein, Lactic, 30 Mesh (200 g), Cystine, L (3 g), Lodex 10 (125 g), Sucrose, Fine Granulaed (72.80 g), Solka Floc, FCC20 (50 g), Lard (245 g), Soybean Oil, USP (25 g), S10026B (Rearch Diets, 50 g), Choline Bitartre (2 g), V10001C (Research Diets, 1 g), Dye Blue FD&C#1 Alum, Lake 35–42% (0.05 g), total amount 7,773.85 g.

Additionally, age- and sex-matched control rats were fed with a standard [low fat diet (LFD)] diet with 10% fat content (D12450B, Research Diets, NJ) with an energy density of 3.82 kcal/g. The caloric information is 10% Kcal fat, 20% Kcal protein, 70% Kcal carbohydrate. Moreover, chow diet contains the following ingredients: Casein, Lactic, 30 Mesh (200 g), Cystine, L (3 g), Sucrose, Fine Granulaed (354 g), Starch Corn (315 g), Lodex 10 (35 g), Solka Floc, FCC20 (50 g), Lard (20 g), Soybean Oil, USP (25 g), S10026B (Rearch Diets, 50 g), Choline Bitartre (2 g), V10001C (Research Diets, 1 g), Dye Yellow FD&C#5 Alum, Lake 35–42% (0.05 g), total amount 1,055.05 g.

During the procedure, both experimental groups were fed ad libitum for 9 weeks. Food intake and body weight were measured weekly. Moreover, fasting glucose was measured by tail incision 1 week before the euthanasia using Accu-check performa device (Roche). Procedures are detailed in Fig. 1.

### Blood collection

In the literature there are several protocols that are suitable for common studies on platelet function. Nevertheless, it is important to choose adequately the best option for blood collection and platelet isolation depending on the planned experiment. In the case of our study, protocols were designed based on several guidelines developed for mice platelets that we modified and adapted to our rat model (Fig. 3a,b)^11,12^.

Rats were anesthetized with Ketamine/Xylazine cocktail (KETAMIDOR (ketamine 100 mg/mL, Richer pharma) and ROMPUR (Xylazine, 20 mg/mL, Bayer) intraperitoneally for all blood collections. The analgesia was tested by loss of reflexes after pinching the rat between the toes and touching their eyes. After that, the abdomen was cut open along the *linea alba*. The gastrointestinal tract was moved to the right side to visualize the vena cava (Fig. 3a).

The needle was inserted into the vena cava in a flat angle between 25° and 45°. Blood was drawn into a 19G 10 mL syringe preloaded with 10% sodium citrate and was aspirated gently until maximal syringe volume was reached or blood flow into the needle ceased (around 6–8 mL). It is important to avoid sucking the vessel wall to the needle.

### Platelet isolation

Methods used in human platelet studies have been adapted during years for animal platelet assays. In our case, we performed a modified protocol based on a practical guide for blood collection and platelet isolation in mice carried out by Aurbach et al.^12^. The platelet isolation protocol was based on a serial of centrifugation steps in order to avoid contaminations with other blood cells and spontaneous platelet activation^31^ (Fig. 3b).

Once the needle was removed from the syringe, around 1.5 mL of blood were transferred from the syringe into 2 mL collection tubes with 150 µL of pre-warmed acid citrate dextrose (ACD, 117 mM sodium citrate, 111 mM glucose, 78 mM citric acid). After that, blood was centrifugated at room temperature for 15 min at 200*g* using a fix angle centrifuge. It is important to highlight that after the first centrifugation step; blood was stood for 5 min vertically in order to take down more efficiently the red blood cells.

PRP from the same individual was pooled and transferred into a 50 mL tube. Prostacyclin (final concentration 1 μM) was then added to the PRP which was centrifugated immediately for 10 min (1,000*g*) at room temperature in a swing out centrifuge. After this step, platelet-poor plasma was aspirated and the pelleted platelets were resuspended in a buffer containing 400 µL of ACD (10%) and 3.6 mL of Tyrodes-HEPES buffer (134 mmol/L NaCl, 0.34 mmol/L Na_2_HPO_4_, 2.9 mmol/L KCl, 12 mmol/L NaHCO_3_, 20 mmol/L HEPES, 5 mmol/L glucose, 1 mmol/L MgCl_2_, pH 7.3) (90%).

Platelet concentration was counted using a Z1 Coulter Particle Counter (Beckman Coulter). 5 µL of the platelet suspension was used to mix with 10 mL of Isoton II. After counting, platelets were centrifugated during 10 min (1,000*g*) at room temperature in a swing out centrifuge in presence of prostacyclin (final concentration 1 μM). Once the pellet was obtained, platelets were diluted with Tyrodes-HEPES buffer to the desired concentration (1 × 10^8^ platelets/mL) and allowed to rest for 30 min at room temperature.

### Flow cytometry assays

In order to validate the quality of platelet isolation protocol, a FACs-related assay was carried out. Moreover, we also measured platelet size by Forward Scatter (FSC) parameter since it is known to be a common indicator of platelet size.

Following the above methodology, 10 µL of washed platelets (5 × 10^7^ cells/mL) was diluted with 200 µL of diluent (Beckman Coulter, USA) and then measured using an Accuri C6 flow cytometer (BD Biosciences). Due to sample limitation, tests were run in duplicate. Data were represented as the geometric mean FSC values and the geometric mean of the % in platelets gate and the % in other cell bloods gate.

### Platelet static adhesion assays

96-well assay plates (Nunc MaxiSorpTM) were coated with collagen Horm (5 µg/mL; Takeda Austria GmbH (Austria)) and rat fibrinogen (100 µg/mL; Enzyme research, USA) over night. Firstly, blocking solution [2% bovine serum albumin (BSA)] was used for 1 h in order to avoid any non-specific adhesion. After 1 h, blocking solution was removed and plates washed with PBS three times. Then, 50 µL washed platelets at 1 × 10^8^ platelet/mL were exposed onto coated plates for 1 h at 37 °C. Unbound platelets were removed by washing the plates three times with Tyrode’s buffer and then adhered platelet were treated with the substrate solution (*p*-nitrophenyl phosphate) for 40 min in agitation. NaOH (3 M) was added and the absorbance (405 nm) was measured with a Tecan Infinite M1000. The assay was measured in triplicate. The results were represented as arbitrary units normalizing the absorbance value with the absorbance value from the negative control (without ligand).

### Western blotting

Western blotting was performed to analyze individual samples (biological replicates) for validation purposes. Platelets were lysed in 2% SDS (final concentration) and following protein quantitation by Pierce 660 nm Protein Assay (Thermo Scientific, USA), proteins were separated by 11% SDS-PAGE. Five micrograms of protein were loaded per lane. The primary antibodies used for immunoblotting were rabbit anti-phospho-SRC (Tyr419) (Invitrogen), dilution 1:1,000 and rabbit anti-SRC pan (Invitrogen). Following washes in TBS-T, membranes were exposed to horseradish peroxidase–labeled goat anti-rabbit (dilution 1:5,000) (Pierce, Rockford, IL), and processed using an enhanced-chemiluminiscence system (ECL, Pierce, Rockford, USA).

### Statistical analysis

Categorical variables from both groups are expressed as mean with standard deviation (SD) and were compared using the Fisher exact test. Differences between groups were analyzed by unpaired *t* test. *p* values of < 0.05 were considered significant.

## Supplementary information

Supplementary Inormation.
