# Supplementary material for: Analysis of platelets from a diet-induced obesity rat model: elucidating platelet dysfunction in obesity
Source: Sci Rep. 2020 Aug 4;10:13104. doi: 10.1038/s41598-020-70162-3 (PMC7403150; doi:10.1038/s41598-020-70162-3)
Supplement: Supplementary file 1 — Supplementary Inormation. [file 41598_2020_70162_MOESM1_ESM.pdf]

Analysis of platelets from a diet-induced obesity rat model: elucidating platelet dysfunction in obesity

María N. Barrachina[1], Luis A. Moran[1], Irene Izquierdo[1], Felipe F. Casanueva[2], María Pardo[3], Ángel García[1].

[1]Platelet Proteomics Group, Center for Research in Molecular Medicine and Chronic Diseases (CIMUS), Universidade Santiago de Compostela, and Instituto de Investigación Sanitaria de Santiago (IDIS), Santiago de Compostela, Spain.

[2] CIBER de Fisiopatología Obesidad y Nutricion (CIBERobn), Instituto Salud Carlos III; Santiago de Compostela, Spain. Department of Medicine, Universidade de Santiago de Compostela, Complexo Hospitalario Universitario de Santiago (CHUS); CIBER de Fisiopatología Obesidad y Nutricion (CIBERobn), Instituto Salud Carlos III; Santiago de Compostela, Spain.

[3]Grupo Obesidómica, Instituto de Investigación Sanitaria de Santiago (IDIS), Xerencia de Xestión Integrada de Santiago (XXS), Santiago de Compostela, Spain.

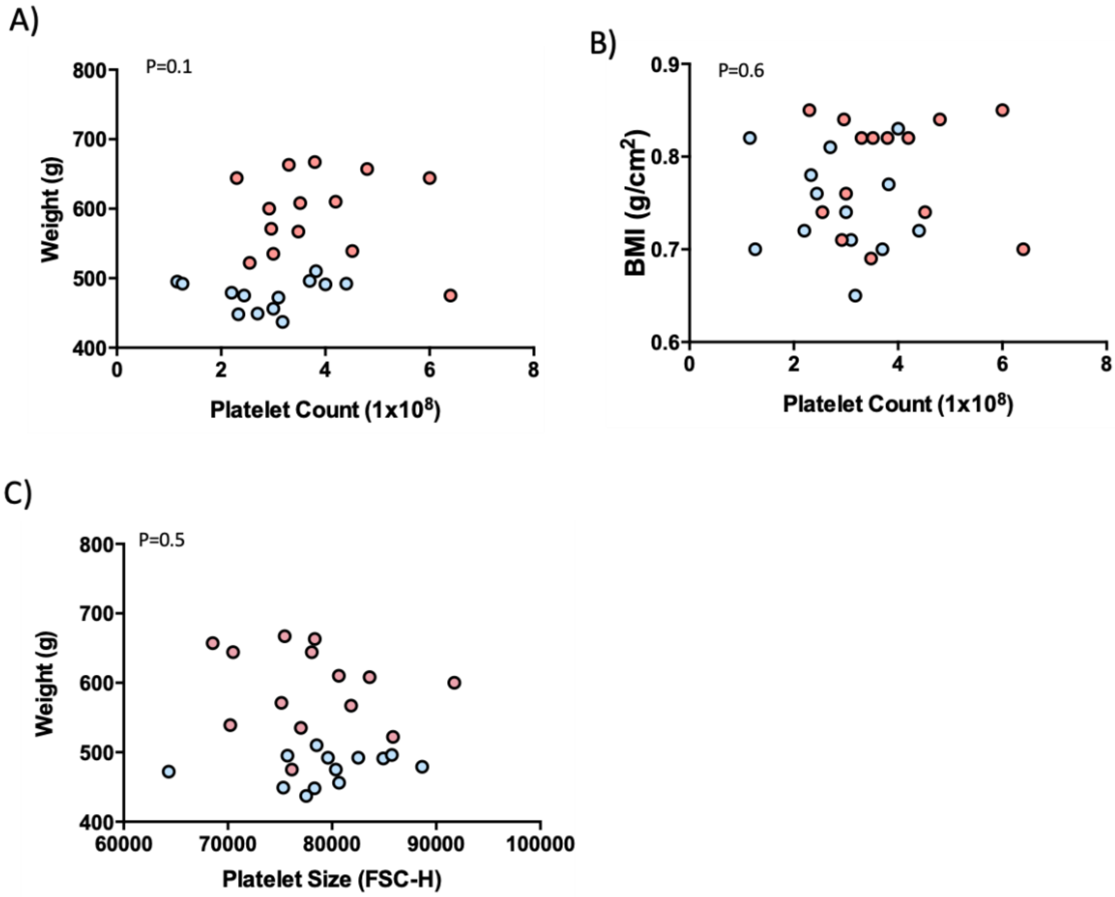

Supplementary Figure 1: Analysis of parameters related to the diet-induced obesity rat model. (A) No positive correlation between platelet count and weight. (B) No positive correlation between platelet count and BMI. (C) No positive correlation between platelet size and weight. Controls (n=13) and DIO rats (n=14) are represented in blue and red color, respectively.

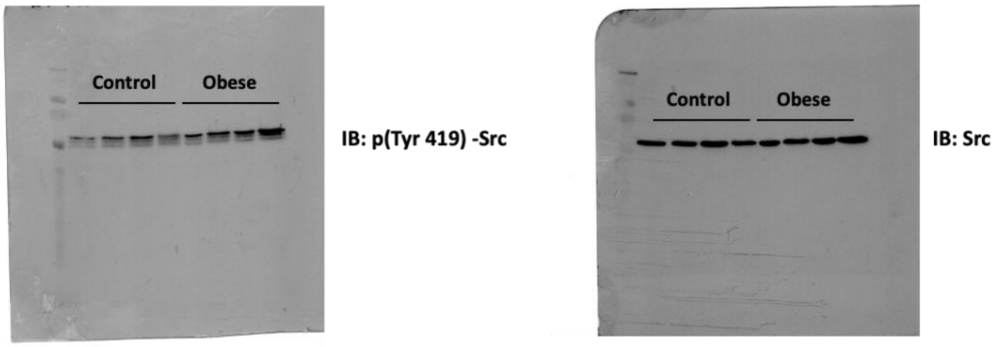

Supplementary Figure 2. Western blot analysis of Src-pTyr$_{419}$ and Src pan protein expression levels in platelets from DIO rat and control samples. Images are representative of the results obtained and show samples distributed in one gel. IB: immunoblot.
